# Feasibility, efficacy, and safety of virtual reality in reducing pain and anxiety during oocyte retrieval: a randomized controlled trial

**DOI:** 10.1186/s12905-026-04272-x

**Published:** 2026-01-15

**Authors:** Li Wang, Juan Liao, Shuozhen Chen, Honggui Wen, Xiaodong Zhang, Hong Luo

**Affiliations:** 1 Center for Reproductive Medicine, Chongqing Health Center for Women and Children, Women and Children’s Hospital of Chongqing Medical University, Chongqing, China; 2 Chongqing Key Laboratory of Human Embryo Engineering and Precision Medicine, Chongqing Health Center for Women and Children, Women and Children’s Hospital of Chongqing Medical University, Chongqing, China; 3 NHC Key Laboratory of Birth Defects and Reproductive Health, Chongqing, China; 4 Nursing School, Zunyi Medical University, Guizhou, China

**Keywords:** Anxiety, Oocyte retrieval, Pain management, Virtual reality

## Abstract

**Background:**

Transvaginal ultrasound-guided oocyte retrieval (TVOR) is a critical and essential step in in vitro fertilization (IVF) and intracytoplasmic sperm injection (ICSI) for the treatment of infertility. However, it is generally considered an uncomfortable and painful procedure. Recent studies have demonstrated the effectiveness of virtual reality (VR) in reducing pain intensity in various acute pain scenarios, including procedural pain. This study aims to evaluate the efficacy and safety of VR interventions in alleviating pain and anxiety among women undergoing TVOR.

**Methods:**

A parallel-group, prospective randomized controlled trial (RCT) was conducted at the Center for Reproductive Medicine of Chongqing Health Center for Women and Children between 1 June 2022 and 30 July 2023. A total of 113 women who underwent TVOR were randomly assigned to either the VR group (*n* = 55) or the control group (*n* = 58), with all participants successfully completing the study. In the VR group, patients wore VR devices to simulate the experience of oocyte retrieval on the preoperative day and watched VR videos during the procedure. The control group received only standard care. The primary outcomes were changes in pain and anxiety scores. The secondary outcomes included changes in physiological parameters, adverse effects, operation cooperation, and pregnancy outcomes in both groups.

**Results:**

Pain scores were significantly lower in the VR group than in the control group both during the procedure (median, interquartile range [IQR]: 3 [2–4] vs. 4 [2–5], *p* < 0.001) and 1 h postoperatively (median [IQR]: 2 [1–3] vs. 3 [2–4], *p* < 0.05). Preoperative anxiety levels measured 10 min before the procedure were also lower in the VR group (mean, standard deviation [SD]: 37.45 [4.92] vs. 39.89 [5.34]; 95% CI: -4.355 to -0.520, *p* < 0.05). Intraoperative physiological monitoring revealed a reduced mean heart rate (mean [SD]: 85.38 [0.87] vs. 90.62 [13.08]; 95% CI: -0.144 to 3.666, *p* < 0.05) and respiratory rate (mean [SD]: 15.64 [2.70] vs. 19.04 [11.40]; 95% CI: 0.168 to 6.422, *p* < 0.05) in the VR group compared with the control group. No significant between-group differences were observed in adverse effects or pregnancy rates (*p* > 0.05 for all comparisons).

**Conclusion:**

This RCT demonstrated that VR significantly reduced pain and anxiety compared with standard care during TVOR, offering a safe, feasible nonpharmacologic adjunct that may reduce opioid needs and shorten the clinic turnover time. These findings provide evidence for integrating VR into routine ART workflows as an alternative, patient-centred intervention, although future multicentre, large-sample studies are needed to evaluate long-term efficacy across diverse clinical settings.

**Trial registration:**

The study was registered in the Chinese Clinical Trial Registry with the code ChiCTR2200060212 on 22/5/2022.

## Background

Transvaginal ultrasound-guided oocyte retrieval (TVOR) is a critical step in the process of in vitro fertilization (IVF) and intracytoplasmic sperm injection (ICSI) [1]. Although considered safe and effective, TVOR is often regarded as the most stressful and painful phase of assisted reproductive technology (ART), involving transvaginal puncture of ovarian follicles to aspirate oocytes for IVF/ICSI. Referred pain after puncturing the vaginal fornices is common and is frequently described as resembling deep menstrual pain [2]. The painful and uncomfortable experience of the procedure can lead to high levels of stress and poor compliance with TVOR procedures. Given its short duration, finding an ideal analgesic method that combines quick efficacy, minimal toxicity, and no negative impact on oocyte quality is essential.

Several analgesia or anaesthesia methods are used for TVOR, including general anaesthesia, epidural or spinal anaesthesia, procedural sedation, nonsedative analgesics, and paracervical blocks [3]. Among these methods, conscious sedation is the most commonly used method for reducing pain during oocyte aspiration [4]. A Cochrane review by Kwan et al. compared conscious sedation with other pain treatments for intra- and postoperative pain but reported that no method was superior [5]. The European Society of Human Reproduction and Embryology (ESHRE) recommendations emphasize incorporating patient preferences and cultural considerations into sedation protocol [6]. However, the lack of standardized guidelines in China’s fast-growing ART field has aggravated inconsistencies in treatment. These challenges highlight the urgent need for nondrug approaches that can provide both pain relief and anxiety reduction without affecting pregnancy outcomes.

A new technology called immersive virtual reality (VR) transports users to a simulated environment, distracting them from the outside world and reducing their perception of pain. It provides an immersive, multisensory, three-dimensional (3D) experience [7, 8]. Growing evidence shows VR’s potential in managing gynaecological pain, including during outpatient hysteroscopy [7], hysterosalpingography [9], and ART procedures such as embryo transfer [10]. A recent randomized controlled trial (RCT) examined VR use during local anaesthesia for TVOR and reported improved analgesia nociception index (ANI) scores without significant reductions in visual analogue scale (VAS) pain ratings [11]. However, the trial did not clearly determine its impact on pregnancy outcomes. Importantly, no existing studies have tested VR’s standalone effectiveness in nonsedated TVOR protocols, and the technology has not been validated in China’s unique clinical and cultural context.

Therefore, we designed an RCT to address three key unmet research needs: (1) measuring the dual effects of VR on both subjective pain (during and after the procedure) and anxiety, as well as objective physiological stress indicators such as heart rate and respiratory variability; (2) evaluating the feasibility of nonsedated outpatient TVOR protocols across clinical settings with different resource capacities; and (3) assessing how the intervention influences final pregnancy outcomes.

## Methods

### Study design

A prospective, parallel group RCT with two parallel groups was conducted at the Center for Reproductive Medicine of Chongqing Health Center for Women and Children between 1 June 2022 and 30 July 2023.

### Study population

Women were eligible if they were aged 20–40 years and were undergoing their first oocyte retrieval for IVF or ICSI. They also needed to have a normal ovarian reserve, defined as a basal FSH concentration < 10 IU/L, an antral follicle count (AFC) > 5, and an anti-Müllerian hormone (AMH) concentration > 1.2 ng/ml. Additionally, they needed to have been using a long agonist regimen for ovarian stimulation and needed to have 6–15 follicles ≥ 14 mm in diameter on the human chorionic gonadotropin (HCG) day. Women were excluded if they had uterine malformations, an endometrial thickness < 7 mm on the HCG day, uterine fibroids > 3 cm, uterine adhesions, chromosomal abnormalities, the use of sedatives or antidepressants, mental illnesses, analgesics taken within the last six hours, or known resistance to virtual reality (usually referred to as motion sickness).

### Ovarian hyperstimulation

During the luteal phase, pituitary downregulation was achieved through daily administration of 0.1 mg triptorelin acetate (Decapeptyl, Ferring) for 18–20 days. Ovarian stimulation was initiated via recombinant follicle-stimulating hormone (FSH) (Gonal-f or Puregon, Merck), recombinant FSH/Luteinizing Hormone (LH) (Pergoveris, Merck), or human menopausal gonadotrophin (HMG, Menopur, Ferring) once pituitary downregulation criteria were met (LH < 5 mIU/ml, FSH < 5 IU/ml, oestradiol, E2 < 50 pg/ml). Gonadotrophin (Gn) dosages were individualized on the basis of the AFC, age, body mass index (BMI), and prior ovarian response. Follicular growth was monitored via ultrasound, and Gn doses were adjusted accordingly. When at least three follicles reached 18 mm in diameter, ovulation was triggered with 250 µg of HCG (Ovidrel, Merck Serono), and oocyte retrieval was scheduled 36 h later.

### Recruitment and randomization

Eligible women were randomly assigned to either the VR intervention or standard care using sealed envelopes prepared the day before oocyte retrieval. Randomization was computer-generated via www.random.org via a random sequence generator. Owing to the visible nature of the VR intervention, participants and intraoperative care providers (surgeons/nurses) could not be blinded to group allocation. However, allocation concealment was maintained until randomization through sequentially numbered, opaque sealed envelopes. Preprocedure and postprocedure outcomes (pain, anxiety) were assessed by research assistants blinded to group allocation. To maintain blinding, patients were instructed to refrain from discussing VR exposure during evaluations, and assessors used standardized scripts to avoid group-specific questions.

### The VR pain control management platform

**Fig. 1:**
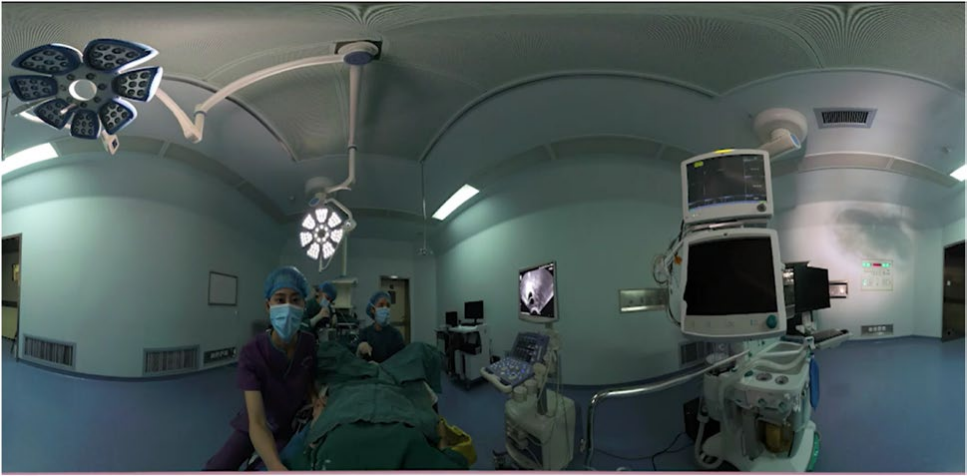
Screenshots of VR video of oocyte retrieval simulation. VR: virtual reality. 3D: three-dimensional

**Fig. 2:**
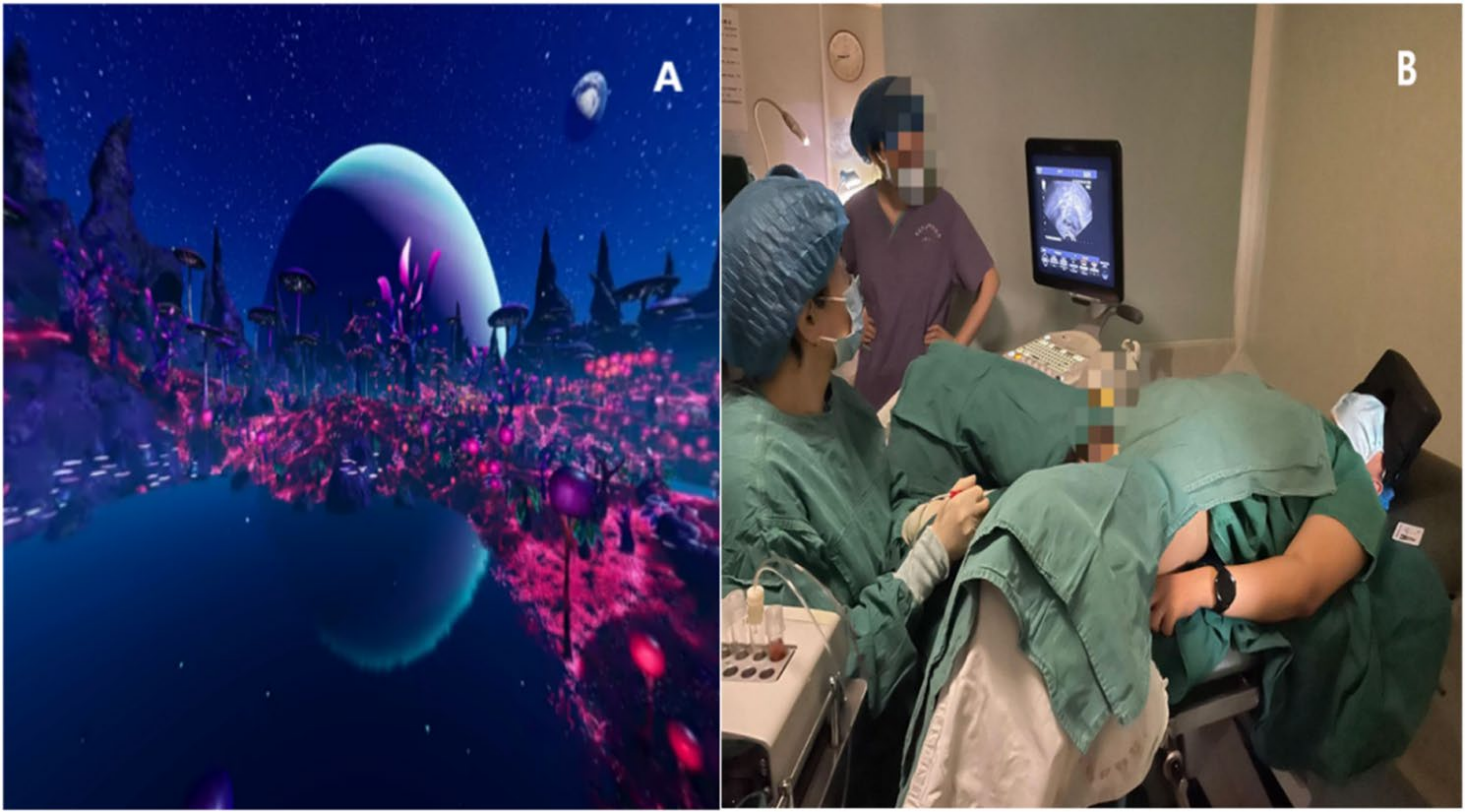
**A**: Screenshots of VR video of Lost Planet Pandora, which was made by the Skyworth VR content production team; **B**: A patient undergoing oocyte retrieval with VR applied to diminish the related-procedure pain and anxiety. VR: virtual reality

The VR pain control management platform was developed by a multidisciplinary team, including clinical experts, health administration specialists, computer programmers, and network engineers. It comprises two modules:

1. Oocyte Retrieval Simulation Experience: A 3D video was created by filming the entire oocyte retrieval procedure, providing patients with a realistic, multisensory simulation of the process through visual and auditory stimuli (Fig. 1).
2. Oocyte Retrieval Analgesia: Patients were immersed in a VR experience titled "Lost Planet Pandora", which was produced by the Skyworth VR content production team (Fig. 2). This module featured vibrant visuals and calming background sound effects to create a relaxing and engaging environment. The participants accessed the VR content via a head-mounted display (Skyworth VR: Applied VR, China).

### Interventions

The standard care group received routine care, including medication guidance, vital sign measurement, health education, and pain care involving soft music during surgery. In the VR intervention group, the same medication instructions and vital sign measurements were given, and pain intervention was carried out through VR. Patients wore VR devices for the simulated experience of oocyte retrieval on the preoperative day and watched Pandora videos during the operation to immerse themselves in a relaxing and pleasant scene, thereby soothing their muscles and reducing the perception of pain. The procedures were performed in a single centre for reproductive medicine by the same doctor and three nurses.

### Outcomes and measurements

**Fig. 3:**
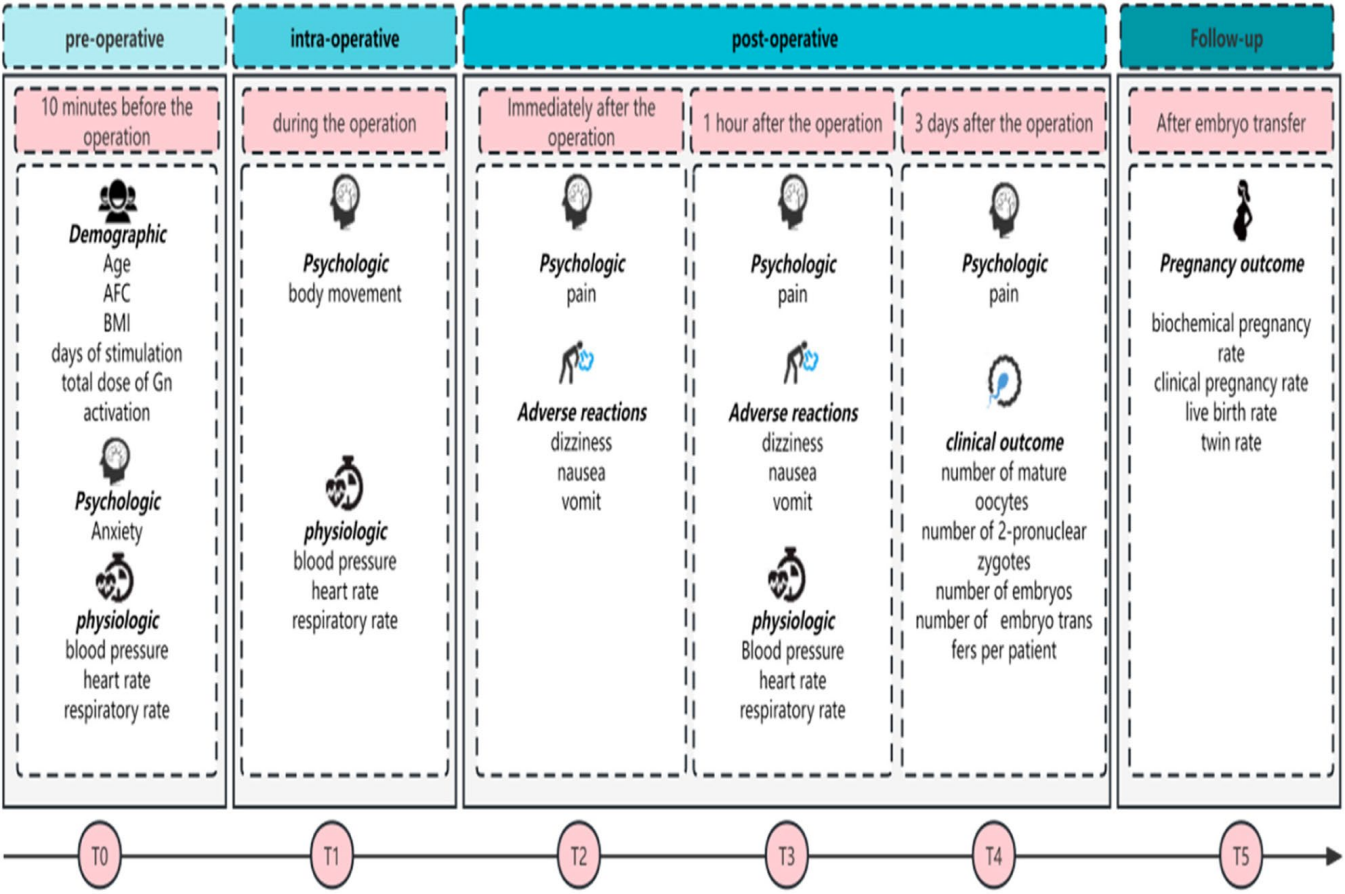
Study design, times and assessments. AFC: Antral follicle count. BMI: Body mass index. Gn: Gonadotrophin

Variables were recorded before and after oocyte retrieval (Fig. 3). The self-rating anxiety scale (SAS) developed by Zung was used to evaluate psychological characteristics. Intraoperative body movement in response to surgical stimulation was recorded and graded as follows: Grade 1, movement of upper limbs; Grade 2, movement of lower limbs below the knee joint, including the knee joint; and Grade 3, movement of the hip. Grade 3 movement could affect the performance of the procedure and potentially cause damage to tissues or organs in the vicinity [12]. Pain was assessed via numeric rating scores (NRS, 11-point scale from 0–10; 0 represents ‘no pain’ and 10 represents ‘worst imaginable pain’) immediately after the operation, 1 h after the operation, and 3 days after the operation. Physiological parameters (heart rate, blood pressure, and respiratory rate) were recorded directly on the patient’s bed, 10 min before the operation, immediately after the operation, and 1 h after the operation. Adverse reactions (dizziness, nausea, vomiting) were recorded immediately after the operation and 1 h after the operation. The numbers of mature oocytes, 2-pronuclear zygotes, and embryos were evaluated as clinical outcomes 3 days after the operation. During the follow-up period, the biochemical pregnancy rate (pregnancy was defined as biochemical when a positive pregnancy test result occurred without any other clinical signs of pregnancy on an ultrasound before week 7), clinical pregnancy rate (defined as an ultrasound-verified pregnancy with a foetal heartbeat), live birth rate (defined as delivery of a live foetus after 22 completed weeks) and twin rate (defined as the proportion of pregnancies confirmed as twin gestations via ultrasound) were evaluated.

### Sample size and statistical analysis

The target sample size for this trial was selected according to Deo et al. [13]. The power calculations were based on a numeric rating score of 6.0 (± 2.62), assuming that a reduction to 3.7 (± 2.66) is expected and targeting a power of 1-β = 90% (alpha = 0.05 for 2-sided tests). The target sample size for this trial was 120 (60 per group) on the basis of our calculation and an estimated 80% participation.

Statistical analyses were carried out via IBM SPSS software, version 26.0. The Kolmogorov‒Smirnov test was used to assess the normality of continuous variables. Continuous variables with a normal distribution are presented as the mean ± standard deviation (SD) and were analysed via an independent sample t test. For continuous variables exhibiting a nonnormal distribution, values are presented as medians and interquartile ranges (IQRs) and were analysed via the Mann-Whitney U test. Categorical variables are denoted by the frequency of occurrence (percentage) and were evaluated via the chi-square test (expected counts ≥ 5) and Fisher’s exact test (expected counts < 5). For all tests, a p value < 0.05 was considered statistically significant.

## Results

### Patient allocation

**Fig. 4:**
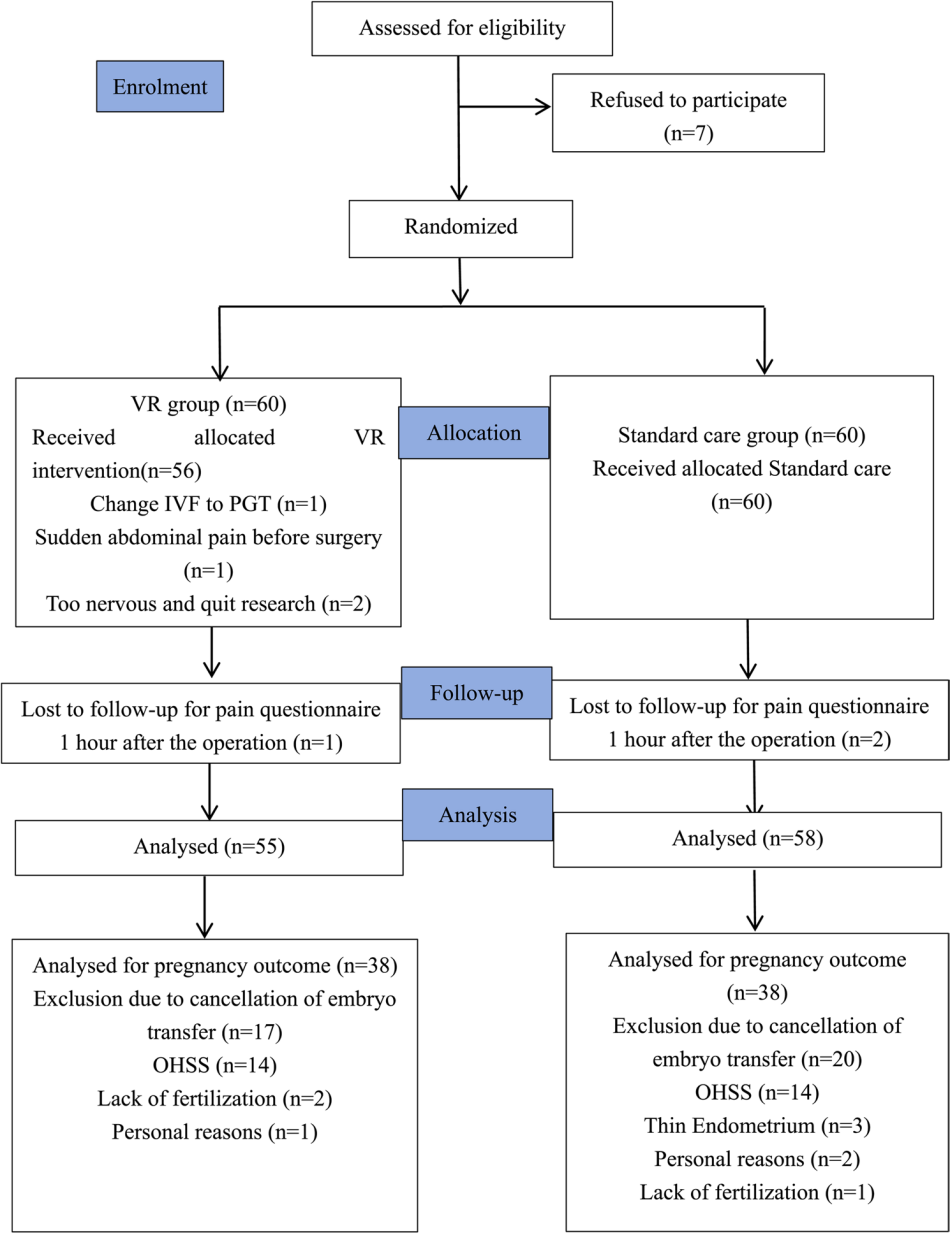
CONSORT diagram showing the inclusion of participants for each stage. VR: Virtual reality. IVF: In vitro fertilization. PGT: Preimplantation genetic testing. OHSS: Ovarian hyperstimulation syndrome

During the recruitment period from June to October 2022, a total of 127 women were assessed for eligibility. Among these, 120 women volunteered and were randomized into either the VR study group or the standard care group, with 60 women in each group. Ultimately, 113 participants (94.2%) completed the study. Figure 4 shows the flow chart of patient recruitment.

### Dropouts

Among the participants, four did not receive the VR intervention: one switched from IVF to preimplantation genetic testing (PGT), one experienced sudden abdominal pain, and two were too anxious to continue. One participant in the VR group was lost to follow-up and failed to complete the pain questionnaire within 1 h of the operation. In the control group, two participants were lost to follow-up. A total of 76 women underwent fresh embryo transfer. The reasons for cancellation included 28 cases of ovarian hyperstimulation syndrome (OHSS), 3 cases of thin endometrium, 3 cases of failed fertilization, and 3 cases of personal reasons. The groups’ dropout rates did not differ significantly from one another (*p* = 0.439) (Fig. 4).

### Patient characteristics

Table 1 displays the baseline characteristics of the participants. There were no significant differences in age, BMI, AFC, stimulation days, or total dose of Gn activation between the standard care and VR groups.

**Table 1:** Demographic characteristics of the participants

Characteristics	VR group (*n* = 55)	Stand care group (*n* = 58)	t	*P* value	95%(CI)
Age (years), mean (SD) ^a^	29.98 (3.50)	31.24 (3.50)	-1.912	0.058	(-2.565,0.046)
BMI (kg/m^2^), mean (SD)^a^	21.57 (1.94)	21.18 (1.70)	1.114	0.255	(-0.285,1.064)
AFC (n), mean (SD) ^a^	8.62 (1.94)	8.71 (2.65)	− 0.193	0.847	(-1.000,0.823)
Day of stimulation, mean (SD) ^a^	10.36 (1.25)	10.09 (1.03)	1.288	0.200	(-0.149,0.704)
Total dose of Gn activation, mean (SD) ^a^	2090.00 (656.51)	2083.40 (651.31)	0.054	0.957	(-237.249,250.455)

*VR* Virtual reality, *SD* Standard deviation, *BMI* Body mass index, *AFC* Antral follicle count, *Gn* Gonadotrophin

^a^: Mean ± SD, independent t test

### Pain and anxiety scores

Table 2 presents the pain and anxiety scores for both groups, along with the procedure length and duration. The median NRS scores for pain during and 1 h after the operation were significantly lower in the VR group than in the standard care group. However, there was no significant difference in pain scores 3 days postoperation. The mean preoperative anxiety scores were also significantly lower in the VR group.

**Table 2:** Comparison of pain, anxiety, vital signs, and operation duration

Variables	VR group (*n* = 55)	Stand care group (*n* = 58)	t/Z/X^2^	*P* value	95%(CI)
Pain, median (IQR) ^a^					
T2	3(2,4)	4(2,5)	-3.688	0.000	/
T3	2(1,3)	3(2,4)	-2.904	0.004	/
T4	1(0,2)	0(0,1)	-1.340	0.180	/
Anxiety, mean (SD) ^b^					
T0	37.45 (4.92)	39.89 (5.34)	-2.519	0.013	(-4.355, -0.520)
Heart rate (per min), mean (SD) ^b^					
T0	99.40 (12.33)	95.98 (12.97)	1.434	0.154	(-1.305, 8.139)
T1	85.38 (0.87)	90.62 (13.08)	1.208	0.043	(-0.144, 3.666)
T3	88.98 (15.20)	88.52 (16.20)	0.157	0.876	(-5.399, 6.328)
Respiratory rate (per min), mean (SD) ^b^					
T0	19.82 (2.74)	19.67 (2.76)	0.282	0.779	(-1.305, 8.139)
T1	15.64 (2.70)	19.04 (11.40)	2.110	0.039	(0.168, 6.422)
T3	18.74 (2.09)	19.95 (8.78)	-1.061	0.291	(-3.761, 1.137)
Sistolic blood pressure (mmHg), mean (SD) ^b^					
T0	119.25 (6.02)	118.91 (6.37)	0.292	0.771	(-1.973, 2.654)
T1	117.53 (4.95)	118.38 (7.15)	− 0.739	0.461	(-3.138, 1.433)
T3	112.95 (3.39)	112.47 (4.85)	0.607	0.545	(-1.086, 2.046)
Diyastolic blood pressure (mmHg), mean (SD) ^b^					
T0	78.85 (3.95)	78.78 (4.22)	0.102	0.919	(-1.446, 1.603)
T1	76.98 (3.54)	76.98 (4.53)	− 0.001	0.999	(-1.521, 1.519)
T3	74.25 (4.29)	74.43 (3.69)	− 0.235	0.815	(-1.664, 1.311)
Procedure duration (min), mean (SD) ^b^	1.91 (1.32)	2.36 (1.45)	-1.734	0.086	(-0.971, 0.065)
Intraoperative body movement, median (IQR) ^a^	1(1,1)	1(1,2)	-2.983	0.003	/
Patients with grade 3 body movement, N(%) ^c^	2 (3.6)	5 (8.6)	0.439	0.242	/

*VR* Virtual reality, *SD* Standard deviation, IQR Interquartile range

T0: 10 minutes before the operation

T1: During the operation

T2: Immediately after the operation

T3: 1 hour after the operation

T4: 3 days after the operation

^a^: Median (interquartile range), Mann‒Whitney U test

^b^: Mean ± SD, independent t test

^c^: *N*(%), Fisher's exact test

### Vital signs

With the exception of heart rate and respiratory rate throughout the procedure, there were no appreciable variations in the groups’ vital signs before, during, or following the procedure (Table 2). The mean heart rate and respiratory rate scores were significantly lower in the VR group than in the standard care group.

### Operation duration

There was no difference in surgical duration between the groups (Table 2). Although the VR group exhibited fewer movements overall, there was no difference in Grade 3 body movements (i.e., hip movement) between the groups.

### Clinical outcomes and pregnancy outcomes

Clinical outcomes, such as the number of mature oocytes recovered, 2-pronuclear zygotes, embryos, embryos transplanted, biochemical pregnancy rate, clinical pregnancy rate, live birth rate, and twin rate, did not differ significantly (Table 3).

**Table 3:** Reproductive outcomes of the two groups

Variables		VR group (*n* = 55)	Stand care group (*n* = 58)	Z/X^2^	*P* value
Treatment outcome	Number of mature oocyte, median (IQR) ^a^	10 (7,13)	11 (8,13)	-0.014	0.989
	Number of 2-pronuclear zygotes, median (IQR) ^a^	7 (5,10)	7 (5,9.25)	-0.747	0.455
	Number of embryos, median (IQR) ^a^	4 (2,6)	4 (2,6)	-0.134	0.893
	Number of embryo transfers per patient, median (IQR) ^a^	2 (0,2)	2 (0,2)	-0.055	0.956
Pregnancy outcome	Biochemical pregnancy rate, *N*(%) ^b^	28 (73.7)	22 (57.9)	0.905	0.342
	Clinical pregnancy rate, *N*(%) ^b^	26 (68.4)	22 (57.9)	2.105	0.147
	Live birth rate, *N*(%) ^b^	25 (65.8)	21 (55.3)	0.881	0.348
	Twin rate, *N*(%) ^b^	7 (26.9)	6 (27.3)	0.000	0.984

VR: virtual reality, IQR: interquartile range

^a^: Median (interquartile range), Mann‒Whitney U test

^b^: *N* (%), chi-square test

### Adverse events

Adverse effects (dizziness, nausea, vomiting) were recorded throughout the study period, and Table 4 presents the results. Although side effects were observed, no significant differences were found between the groups over time.

**Table 4:** Comparison of adverse events between the two groups

Variables		VR group (*n* = 55)	Stand care group (*n* = 58)	X^2^	*P* value
Dizziness and/or nausea and/or vomit, *N*(%) ^a^	T2	2 (3.6)	1 (1.7)	0.612	0.480
Dizziness and/or nausea and/or vomit, *N*(%)^b^	T3	10 (18.2)	10 (17.2)	0.017	0.896

*VR* virtual reality

^a^: *N*(%), Fisher’s exact test

^b^: *N* (%), chi-square test

## Discussion

This study evaluated the impact of a VR-based distraction technique on pain, anxiety, and pregnancy outcomes during TVOR without analgesics or anaesthetics. Key findings include a reduction in NRS pain scores and a decrease in SAS anxiety scores among VR users, providing preliminary evidence for VR as a nonpharmacological analgesic and distraction tool. While no significant difference was observed in pregnancy outcomes, interpretation should be exercised with caution owing to the small sample size for this secondary outcome.

TVOR is known to cause significant pain and anxiety. In this study, the VR intervention significantly reduced pain during the procedure and 1 h postoperation compared with standard care. These results align with those of previous studies demonstrating the efficacy of VR in reducing discomfort during medical procedures. For example, randomized trials have shown reduced pain during colonoscopy without anaesthesia [14], and Estadella et al. reported similar findings during office hysteroscopy [15]. Neuroimaging studies by Hoffman et al. further support these findings, showing that VR reduces activity in pain-related brain regions during painful stimuli, thereby alleviating pain [16]. In our study, the immersive VR environment distracted patients from harmful stimuli, reducing their cognitive focus on pain and promoting relaxation.

Preoperative anxiety, defined as an unpleasant state of tension due to concerns about surgery or the unknown [17], can negatively impact postoperative recovery and delay wound healing [18]. In this study, the VR intervention significantly reduced preoperative anxiety compared to standard care. This aligns with previous research in diverse contexts, including dental surgery [19], labour anxiety [20] and burn wound care [21]. By allowing patients to familiarize themselves with the surgical process through VR one day before the procedure, we reduced their anxiety, improved compliance, and facilitated a smoother medical process.

While systolic and diastolic blood pressure remained unchanged, the VR group presented lower heart rates and respiratory rates during surgery than did the standard care group. This finding suggests that VR interventions can stabilize vital signs by reducing pain levels and sympathetic excitation [22]. However, a study by Fouks et al. in 2022 reported an increased heart rate in the VR group, possibly due to the engaging nature of the VR content [8]. These contrasting findings highlight the need for further research to understand the relationship between VR content and physiological responses.

As the needle punctures through the vaginal wall and explores the eggs in the ovary, the subjects experience pain. Sudden patient movements in response to needle manipulation may increase the risk of tissue or organ damage [23]. In this study, VR intervention reduced intraoperative body movements, improving surgical efficiency and safety. No severe complications occurred, and VR demonstrated the potential to minimize harmful movements through distraction.

VR intervention did not significantly affect reproductive or pregnancy outcomes, including the number of mature oocytes, number of 2-pronuclear zygotes, number of embryos available for transfer, biochemical pregnancy rate, clinical pregnancy rate, live birth rate or twinning rate. These findings suggest that VR does not interfere with assisted reproductive outcomes, whereas reproductive or pregnancy outcomes were collected as secondary endpoints, and the study design was not optimized to evaluate these parameters. Future research with larger cohorts specifically powered for reproductive outcomes is warranted to explore this association in depth.

Adverse effects (dizziness, nausea, vomiting) were reported by 2.65% (*n* = 3) and 17.1% (*n* = 20) of the participants during and 1 h after TVOR, respectively. The rates were comparable between the VR and standard care groups, indicating that VR did not contribute to additional side effects. This may be attributed to several factors: (1) patients prone to VR-related motion sickness were excluded at recruitment; (2) the lightweight VR device used in this study had a short reaction time, minimizing discomfort; and (3) postoperative nausea and vomiting are common after TVOR, often related to elevated oestradiol levels or a history of such symptoms rather than VR [24, 25].

Personalized pain management is essential for oocyte retrieval procedures. Owing to the lack of a standardized anaesthesia protocol, it is necessary to draw on insights from gynaecological pain research to develop individualized strategies. Riemma et al. demonstrated equivalent efficacy of transversus abdominis plane block and wound infiltration for postcesarean analgesia but used fixed-dose local anaesthetics without stratification by patient factors such as BMI or parity [26]. Scheib et al. advocated for enhanced recovery after surgery-based multimodal analgesia to minimize opioid use but overlooked individual adjustments for renal function or sleep apnoea [27]. These gaps highlight the need for future research to prioritize patient stratification (e.g., by BMI, anxiety scores, or surgical complexity) and dynamic protocols. Our findings provide patients with an additional option to choose nonpharmacological analgesia for alleviating pain during TVOR.

In our study, the marked reduction in pain scores with VR (intraoperative NRS score: 3 vs. 4, *p* < 0.001; 1-hour postoperative NRS score: 2 vs. 3, *p* = 0.004) supports its integration as a standard nonpharmacologic adjunct during TVOR. This approach may decrease opioid use in patients concerned with anaesthesia risks, particularly given that anaesthesia-assisted TVOR prolongs treatment time and increases costs. Additionally, some patients have contraindications to anaesthesia. VR adoption could also shorten the clinic turnover time by eliminating postprocedure sedation recovery. Notably, a key strength of this study is the development of a novel VR platform, which includes a 3D video of the TVOR procedure for patient education to alleviate preoperative anxiety.

This study has several limitations that warrant discussion. First, the visible nature of the VR intervention precluded the blinding of participants and surgeons, which may have introduced performance bias. However, we implemented mitigation strategies: an independent nurse, blinded to group allocation, collected all the outcome data to minimize detection bias. Additionally, patient movement during the procedure was evaluated via a standardized 0–3 scale (0 = no movement, 3 = significant disruption), administered and scored by blinded research assistants. This standardized tool, aligned with prior procedural movement assessments (citation needed), helped reduce subjective bias in scoring. Future studies may enhance rigor by using video-recorded procedures for blinded movement analysis. Second, pain was assessed immediately after the procedure rather than during it, which may introduce recall bias. However, a factor that mitigates this concern is that NRS assessment during the operation could disrupt immersion therapy. Future studies could incorporate real-time pain monitoring via devices such as the continuous pain score metre [24] to improve accuracy. Third, the standard care group received soft music, a nonpharmacological intervention that may have influenced outcomes. As highlighted in recent studies [28, 29], music distraction reduces pain and anxiety in gynaecological procedures, which could mask the specific effect of VR. To isolate the unique impact of VR, future trials will develop multimodal nonpharmacological protocols that combine VR, music, and vocal-local techniques with minimal pharmacological agents to minimize drug exposure and reduce reliance on opioids or sedatives [30], which may affect oocyte quality [31]. Additionally, the single-centre design (*n* = 113 patients from a tertiary ART centre) may limit generalizability to clinics, and multicentre RCTs should be conducted to validate the efficacy of VR across diverse populations.

## Conclusion

In conclusion, this study demonstrated the feasibility of VR as a nonpharmacological analgesic for TVOR. VR interventions significantly improved pain relief, reduced anxiety, maintained vital sign stability, and enhanced surgical cooperation during TVOR. These findings underscore the potential of VR as an adjunctive strategy in minimally invasive gynaecological procedures, offering a promising alternative to conventional pharmacological approaches. However, this study was limited by its nonblinded design, postprocedure pain assessment, and music intervention in the standard care group. Future multicentre RCTs with real-time monitoring and multimodal protocols are needed.

## Data Availability

The data that support the findings of this study are available from the authors, but restrictions apply to the availability of these data to protect study participant privacy and so are not publicly available. Data are, however, available from the authors upon reasonable request.

## Abbreviations

TVOR: Transvaginal ultrasound-guided oocyte retrieval
IVF: In vitro fertilization
VR: Virtual reality
3D: Three-dimensional
ICSI: Intracytoplasmic sperm injection
RCT: Randomized controlled trial
ART: Assisted reproductive technology
ESHRE: The European Society of Human Reproduction and Embryology
ANI: Analgesia nociception index
VAS: Visual analogue scale
AFC: Antral follicle count
AMH: Anti-Müllerian hormone
HCG: Human chorionic gonadotropin
FSH: Follicle-stimulating hormone
LH: Luteinizing hormone
HMG: Human menopausal gonadotrophin
E2: Oes tradiol
Gn: Gonadotrophin
BMI: Body mass index
SAS: Self-rating anxiety scale
NRS: Numeric rating scores
PGT: Preimplantation genetic testing
OHSS: Ovarian hyperstimulation syndrome
SD: Standard deviation
IQR: Interquartile range
